# Chordoma: Genetics and Contemporary Management

**DOI:** 10.3390/ijms25115877

**Published:** 2024-05-28

**Authors:** Rupen Desai, Panayiotis E. Pelargos, Ian F. Dunn

**Affiliations:** Department of Neurological Surgery, University of Oklahoma, Oklahoma City, OK 73104, USA; rupendesai922@gmail.com (R.D.); panayiotis-pelargos@ouhsc.edu (P.E.P.)

**Keywords:** chordoma, skull base chordoma, spinal chordoma, sacral chordoma, chordoma genetics

## Abstract

Chordomas, arising from notochord remnants, are rare neoplasms with aggressive growth patterns despite their histologically low-grade nature. This review explores their embryological origins, molecular markers like brachyury, and genetic alterations driving pathogenesis. Diagnosis relies on advanced imaging and biopsy confirmation due to overlapping features with chondrosarcoma. The WHO classification distinguishes conventional, dedifferentiated, and poorly differentiated chordomas, each with distinct prognostic implications. Recent genomic analyses uncovered recurrent mutations in PI3K signaling pathways and chromatin remodeling genes, informing prognostic models. Surgery remains the cornerstone of treatment, though adjuvant radiation complements surgical resection. Although chordomas are generally considered refractory to medical therapy, emerging targeted molecular strategies show potential promise in ongoing trials. This review aims to provide a concise yet comprehensive overview of chordomas, guiding clinicians in diagnosis, treatment, and prognostication for improved patient outcomes.

## 1. Introduction

Chordomas are a distinct category of neoplasms arising from the axial skeleton with aggressive growth characteristics, despite being generally considered to be histologically low-grade tumors derived from notochord remnants [1,2]. Chordomas were first described by Virchow in 1846, though the notochord was not established as a point of origin until 1895 by Ribbert, who demonstrated chordoma induction by puncturing the annulus fibrosis of intervertebral discs in rabbits [3]. The notochord is a midline, rod-like embryologic structure unifying all species of the phylum *Chordata* and expresses key genes driving skeletal development, including *echidna hedgehog* (*ehh*), *sonic hedgehog* (*shh*), *collagen II* (*col2a1*), and *no tail* (*ntl*) [4,5]. In humans, the notochord forms during the third week of development, eventually extending from the sacrum to the sella turcica though progressively replaced by fibrocartilage from the surrounding tissues over the first 1–3 years of life, with notochordal remnants occasionally remaining at the skull base, the odontoid process, intervertebral discs, and the coccyx [6,7,8]. A key molecule in this process is brachyury, a transcription factor that regulates notochord development and thought to be involved in chordoma pathogenesis [9,10].

Today, chordomas have an incidence of just 0.08 per 100,000 population, with an age distribution peaking between 40 and 60 years of age [11]. Importantly, chordomas are most frequently encountered at sites with notochord remnants, with 32% of cases located intracranially, 32.8% in the spine, and 29.2% in the sacrum [11]. While there is a nearly 2:1 male preponderance of spinal chordomas, skull base chordomas have an equal gender distribution [12]. Adolescent (less than 20 years of age) cases are rare, encompassing just 5% of cases, and are most frequently found at the skull base [13].

The WHO classification recognizes three chordoma subtypes: conventional, dedifferentiated, and poorly differentiated chordomas with increasing rates of recurrence and metastases [2]. As we will describe in this review, while generally considered slow-growing malignancies, chordomas are aggressive tumors, both locally and systemically. We will discuss key prognostic and genetic markers used to better understand chordoma aggression and overall survival. Maximal surgical resection is integral to disease control, and although it can be restricted due to location or infiltration around surrounding structures, endoscopic endonasal advances in recent decades have provided additional corridors of safe resection. Both chemotherapeutic and radiotherapeutic adjuvants may be employed in cases of subtotal resection; adjuvant radiation therapy is generally considered the standard of care, though some suggest that radiation may be avoided in the context of gross total resection with a low-risk mutational profile [14].

## 2. Diagnosis

Given the general slow growth of chordomas, symptoms are usually secondary a to mass effect and typically present based on the location of the tumor [2,15]. Lesions affecting the skull base often present with headaches, cranial neuropathy, or endocrinopathies. Chordomas affecting the axial spine can present with localized pain or symptoms of spinal cord and nerve compression, including myelopathy, radiculopathy, and bowel and bladder dysfunction. Finally, chordomas extending into the nasopharynx or cervical spine can present with dysphagia or airway obstruction.

Further diagnostic workup requires advanced imaging; chordomas are typically midline, locally aggressive lesions [16]. Plain radiographs demonstrate lytic bony erosions, often associated with surrounding soft tissue swelling. Computed tomography (CT) shows the extent of the disease and the degree of bony destruction and demonstrates intratumoral calcifications. Magnetic Resonance Imaging (MRI) better characterizes the lesion. T1-weighted images usually show a hypointense lesion, with small hyperintensities representing intratumoral hemorrhage or proteinaceous contents, and mild enhancement with gadolinium contrast. T2-weighted sequences are characterized by marked hyperintensity and frequently have T2 hypointense septations. Key lesions in the differential diagnosis of chordoma include chondrosarcoma, meningioma, myoepithelial carcinoma, glioma, and metastatic carcinoma [12]. Importantly, chondrosarcoma shares similar radiographic features on both CT and MRI; though chondrosarcoma is more likely to have a lateral origin than a midline chordoma, biopsy is often required to differentiate the two.

## 3. Chordoma Genetics and Prognostication

Microscopic evaluation is paramount to chordoma diagnosis and aids in categorizing chordoma into the three WHO subtypes of increasing aggression: conventional, dedifferentiated, and poorly differentiated [2]. Median survival with chordoma is 6.3 years, though is just 16 months in dedifferentiated chordoma. At low power, chordomas are infiltrated and lobulated, separated by fibrous bonds [2,9,17]. Lobules consist of nests of large epithelioid cells with clear cytoplasm and scattered vacuoles in an abundant myxoid matrix, described by the eponym “physaliphorous” from the Greek words for bubble-bearing [9]. Both low- and high-grade nuclear features may be distributed throughout a specimen, and higher-grade regions often have necrosis and mitotic features. Chondroid chordoma is a subtype of conventional chordoma with hyaline cartilage distributed focally or widely throughout the matrix.

A key immunohistochemical marker of chordoma is brachyury, a T-box transcription factor regulating notochord development that serves highly sensitive and specific markers [18,19,20]. Brachyury is amplified in familial chordoma, suggesting its importance as a gain-of-function mutation in chordomagenesis, though genetic duplication in sporadic cases is more frequently associated with whole-chromosome gains [21,22]. Fibroblast growth factor mediates brachyury expression, and its pathway has been implicated in tumorigenesis [23]. Thus, brachyury is a highly sensitive and specific histologic marker of chordoma and is considered diagnostic; other important immunohistochemical markers include epithelial markers (cytokeratin and epithelial membrane antigen—both found in 100% of cases) and S-100 (85.7% of cases) [24]. Ki-67 is a histopathological index of mitotic activity, associated with poorer prognosis when >5% [25].

Genetically, chordomas are notable for the loss of *CDKN2A* and *PTEN* expression and large copy number losses, particularly in chromosomes 1p, 3, 9, 10, 13, 14, and 18; copy number gains are rare [26]. The *CDKN2A* protein p16 is a known tumor suppressor inhibiting cyclin complexes that regulate the G1-S phase of the cell cycle, and loss of expression is a particularly well-established finding in chordoma [27,28,29]. Loss of 1p36 and 9p specifically has been associated with decreased overall survival [25]. Reduced expression of H3K27me3, associated with poor prognosis in other tumors, has also been demonstrated in conventional chordoma [30].

Dedifferentiated chordoma is a more aggressive form of chordoma, representing less than 5% of all chordomas, characterized by frequently abrupt transitions from conventional chordoma to high-grade sarcoma histologic characteristics [31,32]. Dedifferentiated chordoma is generally considered a secondary transformation of conventional chordoma, though there are de novo cases [33,34]. These sarcomatous regions express lower levels of the typical epithelial markers cytokeratin and epithelial membrane antigen, though they have increased vimentin expression [24,35]. A meta-analysis of dedifferentiated chordoma did not reveal significant differences in key demographics of age, gender, tumor size, and tumor location between de novo and secondary dedifferentiated chordoma [36]. The available genetic profiles (five de novo and four secondary) found *p53 as* the most common genetic mutation in both instances; *SMARCA4* is the second most common mutation, though it was only encountered in de novo tumors. Other key mutations of dedifferentiated chordoma from the meta-analysis include *CDKN2A/B*, *IRF2*, *KIT*, *PIK3CA*, *PTEN*, *RB1*, and the *TERT* promoter. A methylation analysis of six skull base dedifferentiated chordoma cases at a single institution demonstrated consistent epigenetic changes in the *TERT* promoter, *MAGEA11*, and *UXT* [32].

Poorly differentiated chordoma is a rare, highly aggressive subtype of chordoma recently recognized by the WHO, most frequently found in adolescents and females, and typically involves the skull base and cervical spine [32,37]. Poorly differentiated chordoma is histologically characterized by cohesive sheets of epithelioid cells with eosinophilic cytoplasm. The cell nuclei are ovoid with focal rhabdoid morphology. The tumors demonstrate either heterozygous or homozygous deletions of the gene *SMARCB1*, encoding an actin-dependent regulator of chromatin [38,39,40].

While initial forays into chordoma genetics evaluated copy number alterations and targeted sequencing, technological analyses in recent decades have permitted whole-genome analyses, providing further information on key mutations. One of the largest such analyses evaluated 104 cases of sporadic chordoma irrespective of underlying histopathology, evaluating a discovery cohort (n = 37) using either whole-genome or whole-exome sequencing, with a subsequent validation cohort (n = 67) [41]. The study found that somatic mutations were rare, with a median of 21 (range 5–76) base-pair mutations and 4 (range 0–47) insertion–deletion mutations per tumor. Of translational relevance, 16% of all chordomas were found to have driver mutations in PI3K signaling pathways, including the genes *PIK3CA*, *PIK3R1*, and *PTEN*. Recurrent mutations were also observed in genes involved in chromatin modeling, including the genes *ARID1A* and *PBRM1* involved in the SWI/SNF complex. Finally, the study found recurrent mutations in the lysosomal trafficking regulator protein, *LYST*, in 14% of the sporadic tumors.

A recent whole-genome analysis of 80 specifically skull base chordomas included 80% histologically classic chordomas, 17.5% chondroid chordomas, and 2.5% dedifferentiated chordomas with a low tumor mutational burden of just 0.53 mutations per megabase [42]. As found in the prior genomic analysis of sporadic chordoma, *PBRM1* was identified as a significant tumor suppressor gene in tumorigenesis. Furthermore, the study found that *PBRM1* was associated with a significantly poorer prognosis when found in conjunction with chromosome 22q deletions, thought to be related to loss of the *SMARCB1* gene affecting the SWI/SNF complex. Other frequently encountered driver mutations in this skull base chordoma cohort included *B2M* and *MAP3K4*. While key driver mutations in other cancer types were found in 17 chordomas, each of these mutations were unique to a specific patient, suggesting significant heterogeneity in skull base chordoma tumorigenesis. An evaluation of the copy number alteration demonstrated gains of chromosomes 1q, 7p, and 7q with deletions of 1p, 3, 4, 9, 10, 13q, 14q, 18, and 22q. Of these, key deletions included 9p21 (in 13.8% of cases), involving the *CDKN2A* tumor suppressor gene, and 3p21 (16% of cases), which includes the chromatin-remodeling genes *PBRM1* and *SETD2*. No significant difference in mutational burden, number of somatic variants, or key driver genetic mutations (*PBRM1/CDKN2A*) were found between the conventional and chondroid tumors, though the patients with dedifferentiated chordoma had elevated tumor mutational burdens, chromosomal abnormalities, and 9p21 deletions. Importantly, the study also evaluated specimens from 11 paired primary and recurrent tumor samples, finding a 30.1% increase in single nucleotide variants, a 43.1% increase in insertion–deletion mutations, and a 43.5% increase in structural variations upon tumor recurrence, though no single genetic alteration was uniformly associated with recurrence.

A recent translational evaluation of chordomagenesis evaluated the effect of individual genetic mutations using *CRISPR* knock-out to screen approximately 18,000 genes in two chordoma cell lines modeling both skull base and sacral chordoma [43]. Similar to prior studies, and serving as a validation of this study, *TBXT* (encoding brachyury) was found to be the top essential gene for chordomagenesis, and *CDKN2A* was also observed as a consistent homozygous mutation. Other key genes included *PTPN11*, *ADAR*, *PRKRA*, *LUC7L2*, *SRRM2*, *SLC2A1*, *SLC7A5*, *FANCM*, *AHR*, *ARNT*, *HEATR3*, *UBIAD1*, *IER3IP1*, *PRKAR1A*, *ZEB2*, *DSCC1*, and *OTUD5*—many affecting similar functional pathways. One such pathway affected, particularly with loss-of-function mutations in *ADAR*, is interferon-stimulated genes, and the study did in fact find elevated levels of interferon expression in chordoma, which enhances chordoma cell viability. Finally, the study identified that several of these genes (particularly *PTPN11*, *EGFR*, and *CDK6*) affect pathways that may be targeted with small-molecule inhibitors.

A meta-analysis of 68 primarily retrospective studies included 3183 unique patients that included 103 biomarkers, defined as molecular (82.5%), histologic (6.8%), radiographic (1%), and physiologic factors (9.7%), for prognostication [44]. The population was primarily (99%) adult, and the tumors included skull base chordomas (50%), sacrococcygeal chordomas (36.6%), and chordomas within the mobile spine segments (13.4%). A univariate analysis found a total of 15 biomarkers associated with either improved progression-free or overall survival, including markers of increased immune activity (elevated CD8+:Foxp3+ ratio, high Immunoscore, PD-1 positive tumor-infiltrating lymphocytes), h-TERT promoter mutations, increased SNF5 expression, c-MET receptor expression, and miRNA-1 expression. Elevated expression of the vast majority of biomarkers (82%) correlated with reduced progression-free or overall survival, with Ki-67 as the most frequently described negative prognostic marker. Importantly, a total of 13 markers were found predictive of both progression-free and overall survival, highly important in a disease like chordoma with high recurrence rates and a long median survival.

Attempts at consolidating chordoma genetic alterations with histologic findings for prognostication have been described. A retrospective analysis of 28 clival chordomas found 32% of tumors with Ki-67 > 5% and 9p loss of heterozygosity or 9p21 deletion in 21% of cases, both strongly associated with shorter survival [25]. Other commonly described genetic alterations were observed, though were not significantly associated with survival. Loss of heterozygosity of the 1p or 1p36 deletion was found in 30% of tumors, and loss of heterozygosity of 10q23 and 17p13 was found in 57% and 52% of tumors, respectively. A 2018 study expanded on these findings, prospectively evaluating 105 clival chordomas at a single institution, specifically evaluating the prognostic ability of Ki-67 and chromosome 1p36 and 9p21 deletions. While all three factors were predictive of prognosis in a univariate analysis, Ki-67 was not independently predictive in a multivariate analysis. Stratifying individual tumors by percentage of cells with chromosomal abnormalities found a high-risk group, where >15% of cells had 1p36 deletions and >25% of cells had 9p21 deletions, and a low-risk group, with <15% of cells demonstrating 1p36 variations and <3% of cells with 9p21 deletions. The group found no significant correlation of these markers with aggressive histologic characteristics, though just 18% of conventional chordomas demonstrated their described low-risk profile, further suggesting the difficulty of predicting prognosis in this limited analysis. A follow-up analysis by the group incorporated surgical outcome and adjuvant radiation into the prior risk stratification, finding that gross total resection improves the prognosis in all genetic groups, gross total resection and radiation therapy are independently associated with an improved prognosis in the high-risk genetic group, radiation therapy is associated with an improved prognosis in the intermediate-risk genetic group only in the context of subtotal resection, and radiation therapy has no benefit in the context of the low-genetic-risk cohort [14].

An analysis of 287 tumors from 111 patients evaluated clinical characteristics, histopathologic factors, and genetic analyses to further prognosticate chordomas [45]. The cohort included both skull base (61.3%) and non-skull-base (38.7%) chordomas of multiple subtypes (60.5% classical, 3.7% chondroid, 28.4% mixed, and 7.3% poorly differentiated). A univariate analysis found that subtotal resection, the absence of postoperative adjuvant proton therapy, poorly differentiated histology, the presence of prominent nucleoli, a high mitotic index (>3/10 high powered field), elevated p53, and elevated Ki67 (>6%) were all significantly associated with reduced overall survival. These factors were developed into a scoring system, dichotomizing into low- and high-risk groups, with excellent correlation with both progression-free and overall survival. In a multivariate analysis, the extent of resection, the absence of adjuvant proton radiation therapy, and histologic grade were independent predictors of progression-free survival. While the molecular analyses were conducted in a limited subset of patients, these analyses were not included within the proposed grading system.

Thus, while there are increasing data and novel technologies to evaluate biomarkers of chordoma recurrence, the heterogeneity of the tumor and a lack of clear understanding of essential clinical, histopathologic, and genetic tumor characteristics is still apparent in our ability to predict disease recurrence and therapeutic response. Some of these key genetic findings are summarized in Table 1. Therefore, further work is essential to consolidate these studies into a clinical decision-making paradigm that may better inform adjuvant therapies.

## 4. Treatment

Despite being described by Virchow more than 150 years ago, management of chordoma remains a clinical dilemma. Although considered a benign disease, the rate of recurrence and aggressive growth patterns of the disease in anatomically restricted regions makes chordoma management difficult. Treatment often starts with aggressive surgical resection and adjuvant radiation therapy, while adjuvant chemotherapy can be considered in refractory cases. Ongoing clinical trials, summarized in Table 2, aim to improve outcomes in chordoma.

### 4.1. Surgery

The extent of surgical resection is one of the most vital characteristics in chordoma management and in reducing recurrence, irrespective of tumor location [46,47,48,49,50,51,52]. Gross total resection is considered the mainstay of chordoma, with en bloc resection representing the gold-standard treatment given the reported risk of tumor seeding [15,53,54]. However, given the notochord origin of the tumor, chordomas often occur in the midline and surround neurovascular structures, increasing the technical complexity of surgery and often eliminating the possibility of en bloc resection. The surgical approach and preoperative decision making are highly dependent on the location of the tumor.

Chordoma of the skull base can be anatomically complex, given the tight proximity to cranial nerves, arteries, and critical structures of the brainstem. Specific approaches depend on the particular characteristics of the tumor and can generally be divided into anterior and lateral approaches, though multiple approaches may be necessary for extensive disease. Anterior approaches (including endonasal and transmaxillary) are midline operations that traverse the clivus for tumor resection, eliminating the need for retraction. Posterolateral approaches include anterior transpetrous, retrosigmoid, and far lateral approaches, and as the category suggests, provide access to a tumor that lies more laterally. A study of 238 patients with skull base chordomas, 73.9% undergoing index surgery, evaluated the results using such approaches [48]. Importantly, the study found that 39.1% of cases showed intraoperative tumor involvement of the dura, increasing the complexity of both tumor resection and reconstruction necessary to avoid postoperative cerebrospinal fluid leaks. In this series, gross total resection was possible in just 11.8% of cases and near total resection in a further 54.2% of cases; prior surgery or radiation therapy significantly reduced the authors’ extent of resection. Common complications associated with surgery include meningitis (8%), CSF leakage (3.8%), cerebral infarction (2.5%), hydrocephalus (1.7%), and death (0.4%). Over the past 20 years, endoscopic endonasal approaches to the skull base have increased in utility, particularly as the risk of postoperative cerebrospinal fluid leak has plummeted with the Hadad-Bassagasteguy nasoseptal flap for reconstruction [55,56,57]. A meta-analysis including 37 studies and 766 patients demonstrated that endoscopic approaches can offer improved rates of gross total resection (61.0% versus 48.1%) and fewer rates of cranial nerve deficits (1.3% versus 24.2%) without significant differences in CSF leak rates compared to open approaches in the modern era; however, there is a steep learning curve in such complex endoscopic endonasal cases, often limiting the approach to specialized centers, and such anterior approaches do not always offer the ideal approach for tumors extending laterally [58,59]. Another meta-analysis evaluating surgical approaches to skull base chordomas included 55 studies and 2453 patients, finding a 33% rate of gross total resection and a 52% rate of subtotal resection, though it did not demonstrate a significant difference in morbidity between the anterior and lateral approaches to the skull base [60]. Given the complexity of surgical resection of skull base chordoma, Brito da Silva et al. designed a grading system to predict postoperative outcomes, generating a score from 2 to 25 based on tumor size, specific location, vascular involvement, the presence of intradural invasion, and prior therapy [61]. In an internal validation cohort, the group found that the scoring system stratified patients into low-, intermediate-, and high-risk cohorts correlating with postoperative outcomes; though such scoring systems have not been verified in larger studies, prognostication systems may help inform surgical decision making and provide an opportunity to guide adjuvant therapy.

Given the risk of chordoma seeding, chordomas affecting the mobile (cervical, thoracic, and lumbar) spine and sacrum are more typically resected in an en bloc fashion than tumors of the skull base. In an effort to better categorize the extent of resection, tumor resection for these regions is delineated by the Enneking classification system, initially designed for musculoskeletal tumors of the appendicular spine, to distinguish wide from marginal resection in the context of tumor invasion [15,49,62]. While surgical margins are preferred, these may not be possible in the spine and sacrum given the proximity to key anatomic structures like dura, the spinal cord, and nerve roots [63].

The sacrum is the most common location for chordoma, representing nearly 50% of all chordomas [11]. A wide margin of resection, including sacrectomy, is well established, and tumor breach is significantly associated with local recurrence (64% recurrence with capsule violation versus 28% without) [64,65,66,67]. In addition to the aforementioned anatomic constraints, wide resection of sacrococcygeal chordoma can be significantly limited by iliac vessels, surrounding musculature, abdominal viscera, and sacroiliac joints; thus, extensive sacral chordomas frequently require a multidisciplinary surgical team to include general surgery, orthopedic surgery, neurosurgery, vascular surgery, and urology to approach the tumor via combined anterior (to establish a plane between the tumor and normal anatomy) and posterior (for completion of the resection) approaches, though posterior-only approaches have been described for limited chordomas [15,68,69]. Resection may entail the sectioning of nerve roots; while the sacrifice of low sacral nerve roots can be limited to a reduction in perineal sensation and alterations in sexual function, the sacrifice of S2/S3 can result in incontinence, while the sacrifice of S1 can also yield deficits in plantar flexion and saddle anesthesia [70,71,72]. Complication rates as high as 25% have been described for sacrectomy, most frequently including wound dehiscence, wound infection, and CSF leak given proximity to the anus, and can be improved with myocutaneous flaps and a prophylactic diverting colostomy [50,68,73].

Chordomas of the mobile spine are much less common than those of the sacrum; the largest study of such spinal tumors was a multi-institutional review including 13 high-volume chordoma centers analyzing 166 patients with chordoma of the mobile spine, finding that approximately 50% of tumors occurred in the lumbar spine and 78% of tumors spanned a single vertebral level [49]. Surgery had a 59% rate of Enneking inappropriate (EI), or intra-lesional, resection; these lesions were more likely to be in the cervical spine, to span multiple levels, and to require adjuvant therapy than tumors with Enneking appropriate (EA) resection. Overall, a 35% rate of local recurrence was observed, with a higher rate in the EI (46%) than EA (16%) cohorts. Although adjuvant radiotherapy was associated with a higher rate of recurrence, this is likely a selection bias because radiation therapy has previously been associated with chordoma control, as we will detail in the upcoming section.

### 4.2. Radiation Therapy

As previously discussed, gross total resection is the gold standard of chordoma management; though the utility of radiation therapy is not well defined, data suggests it does improve outcomes particularly in the context of subtotal resection and recurrent tumors of the sacrum and spine [74,75,76,77,78,79,80]. Although largely considered a radiation-resistant disease, studies have suggested that high-dose photon and proton radiotherapy may improve local control in unresectable disease [78,81]. A retrospective study of 15 patients with sacral chordoma found a mean time to recurrence of 2 years despite en bloc resection, though a trend towards improved survival following immediate postoperative radiotherapy suggests that adjuvant therapy may be beneficial [82]. Given the risk of tumor seeding with intra-lesional resection, neoadjuvant high-dose radiotherapy was suggested in a retrospective study of patients with spinal and sacral chordoma, finding that neoadjuvant radiation seemed to improve local control [83]. A subsequent study of 126 patients with spinal and sacrococcygeal chordoma found that the subset of 28 patients with primary chordoma that received neoadjuvant radiotherapy prior to en bloc resection had no tumor recurrence, and overall neoadjuvant radiation therapy trended towards improved local tumor control [75].

Similar to chordomas of the spine and sacrum, the role of radiation therapy for skull base chordomas is not well defined, and although retrospective analyses suggest that adjuvant radiation might be avoided with specific genetic profiles in the context of gross total resection, the sample size of patients with skull base chordoma treated without adjuvant radiotherapy is limited, and radiation therapy is typically recommended [47,84]. When radiation therapy is administered, proton therapy is generally favored for the treatment of skull base chordoma due to a favorable dosing distribution in the context of the critical locoregional anatomy [85,86]. Although rare, chordoma in the pediatric population has been demonstrated to have excellent prognosis in the context of gross total resection followed by adjuvant proton beam radiation therapy [87]. Given the inaccessibility of proton therapy, a small case series of 12 patients sought to evaluate the efficacy of high-dose fractionated stereotactic radiotherapy as an alternative to proton therapy, finding excellent disease control with 46.9% local control at 2 years [88]. A meta-analysis including 130 patients found non-inferiority of stereotactic radiosurgery alone versus combined stereotactic and fractionated radiotherapy in patients with skull base chordoma [89].

### 4.3. Medical Therapy

Despite broad attempts at medical management, conventional chemotherapies have been unsuccessful in the control of chordoma [90,91,92]. Irinotecan is the only chemotherapy tested in a phase 2 trial, with a response in just 1 of the 15 patients evaluated [93]. More targeted therapy of chordoma is in its early stages, due to recent advancements in understanding its underlying genetic profile. As molecular profiling has suggested alterations in the *KIT* pathway, tyrosine-kinase inhibitors such as imatinib and sunitinib have been trialed, with limited success and the suggestion of some radiographic disease control though without significant changes in disease control [94,95,96]. The overexpression of *EGFR* in a subset of chordomas has led to trialing erlotinib and lapatinib, EGFR inhibitors, with moderate success in promoting partial response or disease stability in advanced chordoma [97,98,99]. Cetuximab, an EGFR antagonist, is currently being assessed for efficacy in advanced chordoma. Multimodal therapies may be necessary; sirolimus, an mTOR inhibitor, was found to have a synergistic effect when combined with imatinib in advanced imatinib-resistant chordoma [95]. An area of increasing research is the effect of the chordoma tumor microenvironment, with the hope that therapeutics may be able to revamp a patient’s immune system to constrain tumor growth [100]. One such class of agents is checkpoint inhibitors, such as nivolumab and ipilimumab, monoclonal antibodies that disinhibit an immune response with remarkable success in systemic tumors and are currently undergoing early-phase trials as both a monotherapy and combination therapy for chordoma. Although success has been observed in both cell lines and early-phase clinical trials, targeted therapies for chordoma require further evaluation to determine their efficacy, and thus effort has primarily been focused in recent years on evaluating surgery with various radiation therapies [101].

## 5. Expert Opinion

Although a pathologically benign disease with an indolent growth rate, chordoma remains a challenging management paradigm. Chordoma often presents late in its disease course with symptoms due to a mass effect from a large lesion encompassing vital neurovascular structures that potentially limit surgical accessibility. Gross total resection unquestionably provides the best surgical outcomes regardless of location, with clear benefits for en bloc resection of the mobile spine and sacrum often made possible with an initial anterior approach to separate the tumor from vascular structures. En bloc resection is not necessary for skull base chordoma, though gross total resection remains of vital importance and may require combined anterior and posterolateral approaches to achieve this goal. Thus, chordoma represents a technically complex surgical disease, and maximal surgical resection is paramount to patient outcomes. The rarity of the disease by nature limits the institutions with sufficient experience to minimize surgical morbidity. In particular, the multidisciplinary surgical approach requires an experienced surgical team, which may include general surgery, neurosurgery, orthopedic surgery, vascular surgery, colorectal surgery, otology, and rhinology experts, depending on a tumor’s specific anatomy. It is likely that low-volume centers do not have the resources or experience to best approach large lesions, and referral to tertiary centers may improve surgical care. Although not well defined at this time, adjuvant radiation therapy seems to provide significant tumor control, even in the context of gross total resection. High-dose radiation therapy seems to provide some tumor control for unresectable lesions. In particular, although limited in availability, proton beam therapy has a favorable profile, with a steep drop-off in radiation allowing targeting near critical structures not possible with fractionated radiation therapy. Further studies are necessary to prove the efficacy of carbon ion radiotherapy and high-dose stereotactic radiation therapy prior to their widespread use for postoperative radiation therapy. Similarly, further studies are needed to better understand the particular molecular profiles that may not require postoperative radiation suggested in prior small cohorts.

Thus, despite over 150 years of improvements in surgical decision making and advances in radiation therapy, the prognosis of this “benign” disease remains poor, with significant morbidity related to both the lesion itself and risks associated with resection itself. Exponential progress through genomic analyses over the past two decades has promoted our molecular understanding of other diseases, unveiling entirely new classes of medical therapies. As a rare disease, such analyses are in their infancy for chordoma, though small-molecule inhibitors and immunotherapeutics like checkpoint blockade therapy have shown promise in translational studies and early-phase clinical trials. Further study is certainly necessary, as the role of chemotherapy is currently extremely narrow and limited to multiple recurrent lesions.

## Figures and Tables

**Table 1:** Key genetic alterations affecting prognosis of chordoma.

Key Genetic Alterations	
***T/TBXT*** (brachyury)	*EGFR*
***CDKN2A/B***	*CDK6*
***PTEN***	*MAP3K4*
***SMARCA4***	*IRF2*
***SMARCB1***	*KIT*
***PBRM1***	*PI3KCA*
***ADAR***	*RB1*
*B2M*	Copy number losses: 1p (**1p36**), 3, 9 (**9p**), 10, 13, 14, 18
*PTPN11*	Copy number gains: 1p, 7p, 7q

**Bold/Underlined** represent strong prognostic alterations.

**Table 2:** Actively recruiting clinical trials for chordoma management.

Clinical Trial Identifier	Study Phase	Intervention
NCT04832620	Observational	Evaluation of imaging parameters to predict chordoma and chondrosarcoma treatment response
NCT05286801	Phase I/II	Tiragolumab/atezolizumab for treatment of refractory *SMARCB1/SMARCA4*-deficient tumors
NCT05407441	Phase I/II	Tazemetostat and nivolumab/ipilimumab for treatment of *SMARCB1/SMARCA4*-deficient tumors
NCT04416568	Phase II	Nivolumab/ipilimumab for treatment of *SMARCB1/SMARCA4*-deficient tumors in children
NCT05041127	Phase II	Cetuximab (anti-EGFR) therapy in advanced/unresectable chordoma
NCT05861245	Phase II	Hypofractionated proton beam therapy for chordoma/chondrosarcoma
NCT06140732	Phase II	Apatinib (anti-PD1) + Camrelizumab (anti-VEGFR) in advanced chordoma
NCT02838602	NA	Randomized carbon ion versus standard radiotherapy for radioresistant tumors
NCT06029218	NA	Comparison of 1- versus 2-beam proton beam therapy
NCT05707767	NA	Analysis of surgical techniques for resection of spinal chordoma
NCT02986516	NA	Surgery + adjuvant XRT versus XRT alone in sacral chordoma
